# Exploring indirect effects of a classic trophic cascade between urchins and kelp on zooplankton and whales

**DOI:** 10.1038/s41598-024-59964-x

**Published:** 2024-04-29

**Authors:** Lisa Hildebrand, Solène Derville, Ines Hildebrand, Leigh G. Torres

**Affiliations:** 1 Geospatial Ecology of Marine Megafauna Laboratory, Department of Fisheries, Wildlife & Conservation Sciences, Marine Mammal Institute, Oregon State University, Newport, OR USA; 2 UMR ENTROPIE (IRD-Université de La Réunion-CNRS-Laboratoire d’excellence LabEx-CORAIL), Nouméa, New Caledonia

**Keywords:** Ecology, Ecosystem ecology

## Abstract

Kelp forest trophic cascades have been extensively researched, yet indirect effects to the zooplankton prey base and gray whales have not been explored. We investigate the correlative patterns of a trophic cascade between bull kelp and purple sea urchins on gray whales and zooplankton in Oregon, USA. Using generalized additive models (GAMs), we assess (1) temporal dynamics of the four species across 8 years, and (2) possible trophic paths from urchins to kelp, kelp as habitat to zooplankton, and kelp and zooplankton to gray whales. Temporal GAMs revealed an increase in urchin coverage, with simultaneous decline in kelp condition, zooplankton abundance and gray whale foraging time. Trophic path GAMs, which tested for correlations between species, demonstrated that urchins and kelp were negatively correlated, while kelp and zooplankton were positively correlated. Gray whales showed nuanced and site-specific correlations with zooplankton in one site, and positive correlations with kelp condition in both sites. The negative correlation between the kelp-urchin trophic cascade and zooplankton resulted in a reduced prey base for gray whales. This research provides a new perspective on the vital role kelp forests may play across multiple trophic levels and interspecies linkages.

## Introduction

Consumer-mediated trophic cascades are extensively documented in ecology, where the loss of an apex predator from a system has cascading influence downward along a trophic chain^1–3^. The number of trophic linkages in the chain (odd or even) determines whether the top-down influence on lower trophic levels is positive or negative, respectively^4^. This influence can include changes in abundance, distribution, or productivity of lower trophic levels, including autotrophs. Trophic cascades have been reported worldwide in diverse ecosystems^5^, yet the indirect effects of trophic cascades to predators remain largely understudied^6^.

Kelp forests are dynamic and vital ecosystems that have been extensively researched, including in the context of consumer-mediated trophic cascades (e.g.^1,7^). Kelps are foundational species that provide critical habitat, refuge, and food to numerous marine species due to the high rates of primary production within kelp forests^8^. They require cold, nutrient-rich waters to grow ^9^, and while kelps are resilient to short-term warming events^10^, they are at risk of extreme climatic events^9,11^, such as marine heatwaves (MHWs).

Kelp forests can transition to sea urchin barrens, and back again, in phase shifts^12,13^. These shifts usually occur after a change in sea urchin grazing intensity, which can result from changes in sea urchin predator abundance due to human-induced perturbations to the ecosystem, such as overfishing and climate change^14,15^. In some systems, phase shifts can be mediated by urchin predators either through direct consumption (e.g., density-mediated^1^) or their presence causing urchins to behave cryptically (e.g., trait-mediated^16^) by hiding in crevices and feeding passively on detrital matter and drift algae. The reduction or complete loss of urchin predators from a system releases urchins from predation pressure, which can trigger a trophic cascade^1^. In the absence of predation risk or following a reduction in kelp abundance, urchins may switch their foraging behavior from being passive detritivores to actively grazing on kelp, which can lead to a phase shift toward a sea urchin barren^17^. The loss of kelp has direct effects to other species that occupy kelp habitats and rely on it for resources, shelter, and important life-history cycles^18^.

Canopy-forming kelp forests have declined in density and abundance in areas along the North American west coast since the mid-2010s^17,19^. Several co-occurring factors contributed to these declines, including a record-breaking MHW which caused nutrient-poor, warm water conditions to persist in the northeast Pacific Ocean from 2014 to 2016^20^. Furthermore, the onset of sea star wasting disease (SSWD) in 2013, which may have been facilitated by anomalous warm ocean temperatures, affected over 20 sea star species^21^. The predatory sunflower sea star (*Pycnopodia helianthoides*) was particularly susceptible with population declines of 80–100% across a ~ 3,000 km range from Mexico to Alaska^22^. In some regions of North America, the loss of this urchin predator released purple sea urchins (*Strongylocentrotus purpuratus*) from predation pressure, leading to increases in purple sea urchin populations^17,19^. Furthermore, in regions where sea otters currently do not exist and therefore sunflower sea stars were the only urchin predator, suboptimal ocean conditions (warm water and low nutrients) depressed kelp recruitment and growth, resulting in less detrital drift kelp for urchins to passively feed on, requiring them to switch to active herbivory on kelp to satiate their hunger^17^. These factors resulted in ecosystem shifts from productive kelp forests to unproductive urchin barrens, which have shown no signs of recovery^17,23^. There is concern that shifts to urchin barrens may become an alternative stable state of the subtidal ecosystem from which kelp cannot locally recover if urchin predators are absent^24^.

The Pacific Coast Feeding Group (PCFG) of gray whales (*Eschrichtius robustus*) spend their summer feeding season in coastal, nearshore waters between northern California, U.S.A and northern British Columbia, Canada^25^. PCFG whales have a broad diet and employ a range of foraging tactics in different benthic habitats to obtain their prey^26^. Mysid shrimp (*Neomysis rayii* and *Holmesimysis sculpta*) are energetically profitable and consistently available prey items of PCFG whales^27^. These mysid species are found strongly associated with kelp forests and canopies within the PCFG range^28–30^. Mysids associate with kelp-dominated reef habitat for a number of reasons. The high productivity in kelp beds offers mysids a wide variety of prey items to satisfy their omnivorous diet, which includes kelp zoospores^31^. Mysids may be retained in kelp forests where current velocities can be up to one third slower than outside^32^. Furthermore, upwelling shadows and nearshore fronts surrounding reef habitats can support such retention^33,34^. Finally, mysids may have a particular affinity for associating with the kelp canopy as potential protection from predators^35^. PCFG whales forage in kelp habitats throughout their foraging range, likely targeting mysid shrimp^26,29,30,36^. PCFG gray whale body condition varies annually^37,38^ but has progressively declined in recent years [37; Torres, unpublished data]. Although the cause of this decline is unknown, it has been speculated that variability in prey quantity and quality caused by kelp forest declines may be affecting gray whale foraging success^30^.

The Oregon coast (U.S.A) comprises ~ 34% of the ~ 1400 km long range of the PCFG, where the predominant urchin predators are sunflower sea stars since sea otters (*Enhydra lutris*) have been absent from Oregon waters for over a century^39^. Hence, following the SSWD event that completely decimated sunflower sea star populations locally^22^, a trophic cascade may have begun as purple sea urchins were released from predation pressure. Similar ecosystem shifts were documented in northern California^17^, which is biogeographically similar to our study site located in Port Orford along the southern Oregon coast. We monitored gray whale foraging ecology in Port Orford for 8 years (2016–2023) following both the SSWD and MHW events and analyzed our spatially explicit dataset on habitat, prey, and predators to address the hypothesis that this trophic cascade indirectly affects zooplankton prey and whale predators. We hypothesize that purple sea urchin coverage increased in our study area as a result of urchins being released from predation pressure following the 100% decline of sunflower sea stars in the region^22^. We predict that bull kelp (*Nereocystis luetkeana*), which was likely already depressed due to persistent warm and nutrient poor ocean conditions, suffered declines in frond and stipe condition due to active herbivory by urchins. We posit that the decline in kelp condition ultimately results in a loss of kelp canopy density as the fronds make up the canopy. We hypothesize that zooplankton (primarily dominated by mysid shrimp in this area, ~ 85% of community; Torres, unpublished data) abundance declined in part due to this reduction of kelp canopy, which zooplankton rely on for habitat and productivity. Finally, we predict that gray whale foraging time was reduced in this study area for two reasons: (1) the decline in abundance of their zooplankton prey base and (2) the loss of kelp canopy, which we hypothesize gray whales may use as a cue to locate zooplankton given their consistent range-wide association with kelp^26,29,30,36^. While kelp-associated trophic cascades are commonly described in the literature, the link to zooplankton, which are a critical food source for whales and other nearshore species that rely on kelp ecosystems, have not been previously described.

## Methods

### Field methods

**Figure 1:**
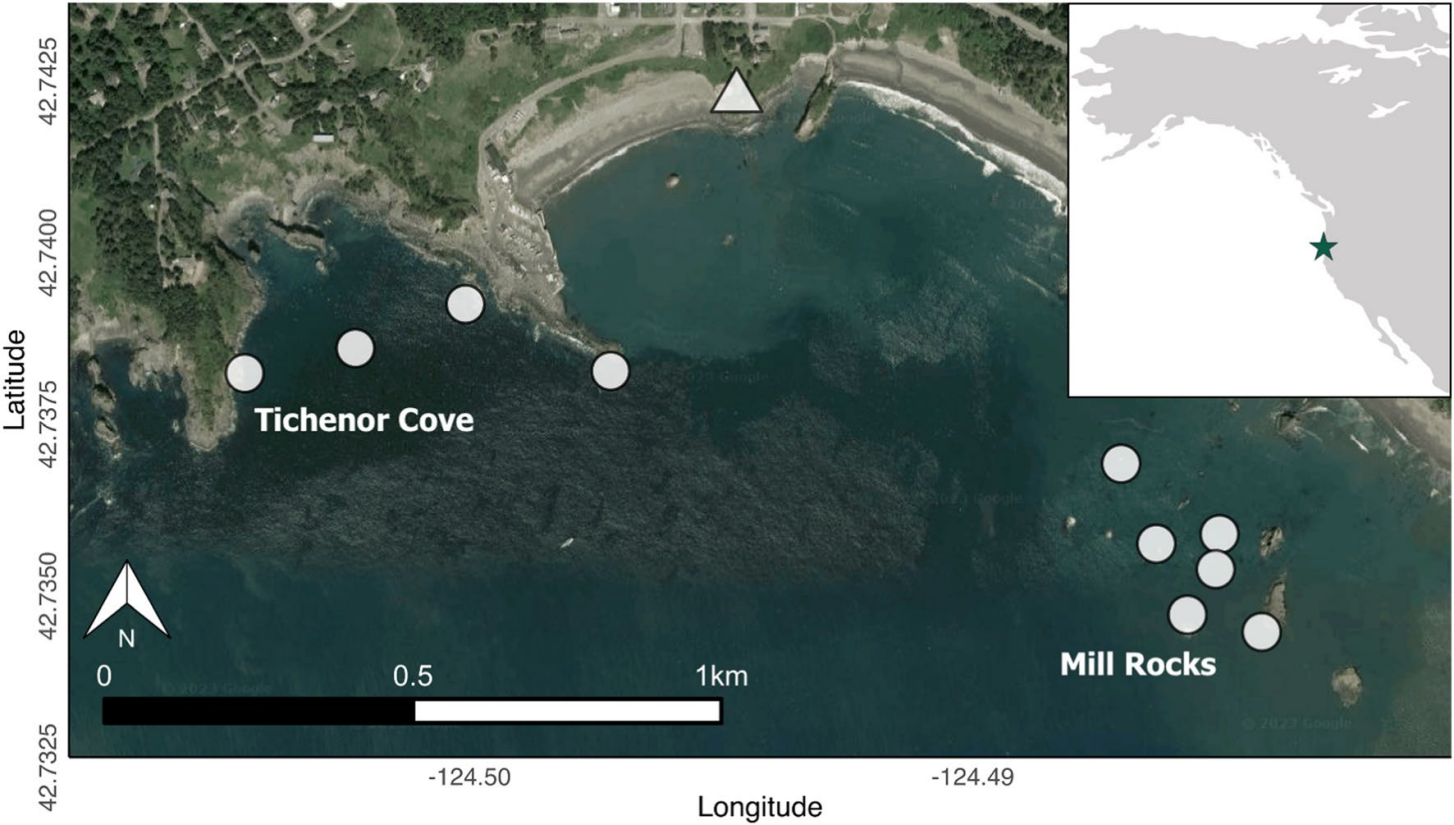
Map of Port Orford, USA study area showing the 10 kayak sampling stations (white circles) within the two study sites (Tichenor Cove and Mill Rocks). The white triangle represents the cliff top location where theodolite tracking of whales was conducted.

Data for this study were collected as part of a larger study on fine-scale gray whale foraging ecology ^30^ near the coast of Port Orford (Fig. 1), Oregon, U.S.A, between July and August from 2016 to 2023. Field sampling occurred during daylight hours, beginning in the morning (~ 06:30 h), and suitable weather conditions (wind < 10 knots, swell < 1 m, visibility > 3 km). Field sampling ended when ocean conditions compromised accurate data collection (Beaufort sea state > 3) or when the whale observation team had surveyed for 8 h. We non-invasively tracked gray whale movements using a theodolite (Sokkia model DT210) in two study sites (Mill Rocks [MR] and Tichenor Cove [TC]) that are viewable from a cliff top location (elevation = 33 m). Photographs were taken using a Canon EOS 70D camera to identify individual gray whales based on unique markings. When more than one whale was present in the study sites, theodolite tracking was paused to ensure that the same individual was reliably tracked. However, this situation occurred rarely and represents only a small portion of the dataset (2.3 h out of 890 h of total tracking, or 0.26% of the time). Concurrent to tracking whales, we conducted daily assessments of habitat and prey availability with a tandem kayak navigating to 10 target locations within MR (*n* = 6 locations) and TC (*n* = 4 locations), where the seabed substrate was composed of rocky reef (Fig. 1). These stations were selected based on previous observations of whale foraging in the area and to ensure an optimal sampling of the patchy reef and kelp forest habitats within a day^40^. The minimum and maximum distances between sampling stations were 57–356 m in MR and 112–595 m in TC, which aligns with the fine-scale variations that are expected in these habitats. The kayak was launched at the start of the day (~ 06:45 h) and all stations were sampled once (unless ocean conditions deteriorated, in which case sampling was aborted). At each sampling station, we (1) used a Secchi disk to measure water clarity and (2) performed a paired GoPro (Hero4) and Time-Depth Recorder (TDR; Solinst Levellogger 3002 F100/30) drop. The paired system was lowered to the bottom, where it rested for approximately 10 s, before it was pulled up at a consistent speed (0.1 ± 0.05 m/s) using a downrigger. The kayak team maintained position as best as possible, but drift from the target station occasionally occurred and the coordinates of the actual sampling station were always recorded with a hand-held GPS. Therefore, only data from videos that were obtained within 10 m from the target location coordinates were included in this study to ensure that repeat measurements were representative of that location. The GoPro videos were used to assess kelp condition, exposed urchin proportion, and zooplankton abundance. These non-traditional methods of zooplankton and habitat assessment were applied because these nearshore, shallow reef habitats that contain kelp limit effective use of traditional methods such as echosounders.

### Species occurrence metrics

Videos were processed to obtain a relative measure of bull kelp condition, and to quantify exposed purple sea urchin coverage and zooplankton relative abundance, while theodolite tracklines were analyzed to determine gray whale foraging time at target stations. Videos were selected for processing and analysis if they met the following criteria: (1) visibility of ≥ 2 m (determined with Secchi disk), and (2) if sampling occurred within 10 m from the target station coordinates. After removing all drops that did not meet the selection criteria, 741 out of 1,297 GoPro videos were used to derive urchin, kelp, and zooplankton occurrence metrics across the eight years. A single analyst processed each component of the data (kelp condition, urchin coverage, zooplankton abundance) to ensure consistency. Additionally, a second analyst processed a subset of each component of the data blindly to ensure robustness of methods. We randomly subsampled a total of 120 images and videos from all eight years (*n* = 15 from each year), and measurements between the two analysts were compared by calculating the coefficient of variation (CV%) for these 120 subsamples. Our video processing methods described below were specifically designed to accommodate our study design and enable use of eight years of standardized data collection. While our methods are unconventional and do not measure absolute levels of urchin density or kelp condition, our approach derives accurate relative measurements that enable us to assess correlations between two co-occurring taxa in video data initially collected for other purposes^30^. We validated our unconventional methods against more traditional methods for assessing urchin and kelp density (see ‘Validation of non-traditional methods’ Section below).

#### Exposed purple sea urchin coverage

**Figure 2:**
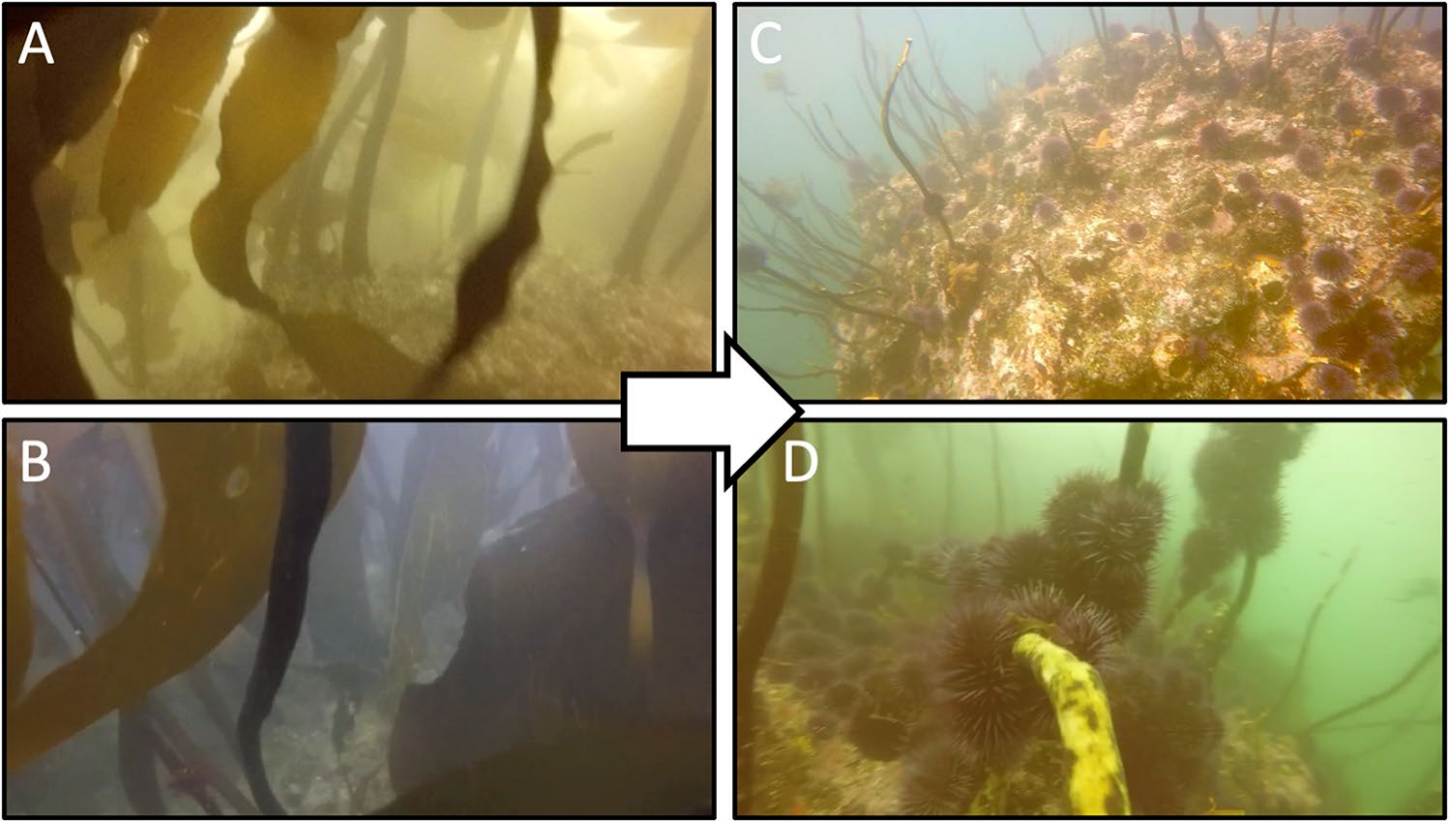
Example images from GoPro videos at two sampling stations in Port Orford, Oregon illustrating observed ecosystem shifts: pre-impact (**A** & **B**) and post-impact (C & D) conditions.

We estimated exposed purple sea urchin (hereinafter ‘urchin’) coverage at each sampling station by first selecting the still image with the greatest amount of suitable urchin substrate from each GoPro video. We define suitable urchin substrate as any visible rocks and/or kelp stipes in a reef. We do not consider sandy bottom or corals as suitable urchin substrate since urchins were not observed on these substrates in the eight years of this study (Fig. 2). The entirety of the GoPro video was reviewed (down and up cast) to select the still image that contained the greatest amount of suitable urchin substrate as well as optimal clarity relative to the rest of the video. Next, images were imported into ImageJ. For still images that contained urchins, solid, white circle shapes were drawn on top of fully visible urchins. These white circles provided strong color-contrast relative to the rest of the image, which allowed us to create an automated macro in ImageJ that used color and contrast thresholding to isolate the white circles that represented the urchins and then calculated the pixel area of the white urchin circles. If only half or less than half of an urchin was visible, no white circle was placed on it and therefore it was not counted. Next, we calculated the number of suitable urchin substrate pixels in each still image to quantify the pixel area where urchins could have occurred in the image. To do this, we traced all suitable urchin substrate in ImageJ and filled these traces with a blue color. Once again, the blue color provided strong color-contrast relative to the rest of the image, which allowed us to create a second automated macro in ImageJ that used color and contrast thresholding to isolate the blue traced area that represented substrate where urchins could have been and then calculated its pixel area. For example images of these steps, see Fig. S1. Once this image analysis was completed, then the urchin coverage was calculated for each station video by dividing the number of urchin pixels by the number of urchin pixels plus the number of suitable urchin substrate pixels. We then calculated the mean urchin coverage across all sampling stations within each site on each day.

Urchin monitoring in reef systems typically occurs by counting urchins in an area of known size (e.g., 1 m^2^ quadrat) or along a known distance (e.g., 30-m transect) in order to derive density estimates. Our GoPro camera setup did not contain lasers and we were therefore unable to quantify absolute area assessed. However, in order to ensure consistency in the substrate area assessed at each sampling station, we implemented a minimum threshold of relative substrate that needed to be visible in order for a station to be included in the analysis. We did this by only including videos of sampling stations where enough substrate was visible to accommodate at least 10 urchins. Given that the average size of a purple sea urchin is 7.5 cm^41^, we assessed on average 0.1 m^2^ at each station (range: 0.06–0.88 m^2^).

#### Kelp condition

Bull kelp (hereinafter ‘kelp’) condition at each daily sampling station was assessed qualitatively by viewing the entire GoPro video cast from a given sampling day (rather than assessing kelp health in the single still image in which urchin coverage was assessed). This method was chosen due to the fact that kelp could not be accurately described from a single still image within a video. The five kelp health categories were as follows: all damaged (AD), mostly damaged (MD), mostly health (MH), all healthy (AH), and no kelp (NK). Reference videos (see video files in the Figshare data repository) were used to ensure consistency in kelp health category assignments. The presence of kelp and the condition of both the kelp fronds and stipes were considered to assign one of these categories. AD was assigned if all (100%) kelp fronds and/or stalks were visibly damaged due to urchins (e.g. urchins actively feeding on stalks or extreme fraying of kelp fronds with many urchins seen nearby). MD was assigned if kelp frond and/or stalk damage was predominant (> 50% of kelp plants seen), however if some healthy kelp (e.g. big bull kelp plants with long, intact fronds) were also visible. MH was assigned if most kelp fronds and stalks were long and intact (> 50% of kelp plants seen), however if some unhealthy kelp (e.g. urchins actively feeding on stalks or extreme fraying of kelp fronds with many urchins seen nearby) were also visible. AH was assigned if all (100%) kelp stalks and/or fronds in the video were long and intact. NK was assigned if no part of a kelp plant was visible in the entire video. For analysis, these kelp condition categories were converted to a numerical score: NK = 1, AD = 2, MD = 3, MH = 4, AH = 5. We calculated the mean kelp condition across all sampling stations within each site on each day.

The loss or decline of kelp in a system is typically quantified through changes in kelp forest canopy extent or density. In our study system, there are no contemporaneous measures of kelp forest canopy extent at the daily scale. Furthermore, while we considered counting the number of stipes at each station from the GoPro videos, we would not have been able to reliably count kelp stipes to assess density, given that the GoPro camera spins around considerably during the upcast. Therefore, we developed a qualitative method to categorically score kelp condition. Nevertheless, we posit that our kelp condition metric is representative of the overall health of a kelp plant, which ultimately has an influence on the density of the canopy. Since the fronds of the kelp plant are what form the canopy, if the fronds are damaged or absent, then the canopy becomes reduced or disappears.

#### Zooplankton relative abundance

As described by^30^, still images were extracted every 5 s from the upcast of the GoPro videos and divided into a 3 × 3 cell grid (each cell measured 533 × 953 pixels). Each grid cell was assigned a score from 0 to 5 according to the relative amount of zooplankton in the grid cell using standardized methods based on reference images (with obscured and low visibility cells assigned NA values; see Fig. S2). Mean scores across cells were calculated per still image and then summed per video to obtain a relative zooplankton abundance for each daily sampling station. We then calculated the sum of relative zooplankton abundance across all sampling stations within each site on each day.

In addition to conducting GoPro drops at each sampling station, the kayak sampling team also drops a zooplankton net to collect a representative sample of the prey community. However, this sampling only began in 2017, therefore we used zooplankton relative abundance values calculated from the GoPro videos as these spanned the entire study period (2016–2023). Furthermore, using the GoPro videos to quantify zooplankton relative abundance allowed us to assess a larger spatial area and field of view as opposed to capturing prey from a point location through a vertical tow, which mysid shrimp effectively evade^42^.

#### Gray whale foraging time

Theodolite locations of whale surfacings were analyzed and behaviorally classified using RST^30,43^ into transit, search, or feed states. The number of feed and search points within each site on each day were summed to estimate foraging time in minutes.

### Validation of non-traditional methods

We validated our non-traditional methods of quantifying purple sea urchin coverage and kelp condition, against more traditional methods for assessing urchin and kelp density. The results of these validations can be found in the Supplementary Information (Figs. S3 and S4, Text S2).

#### Exposed purple sea urchin coverage

We validated our urchin coverage method using purple sea urchin densities derived from stereo imagery acquired by the Oregon Department of Fish and Wildlife (ODFW) in a separate sea urchin-specific video survey (see Supplementary Text S1 for further details about the imagery). We applied our urchin coverage method (described above) to 14 images provided by ODFW (ODFW, unpublished data). We then counted the number of urchins visible in each image and divided this by the number of suitable urchin substrate pixels that we calculated using our method. We then compared our urchin values to ODFW’s urchin densities (m^2^). We calculated the Spearman’s correlation coefficient to statistically compare the two methods.

#### Kelp condition

We validated our kelp condition scores against canopy area values derived from Landsat satellite imagery produced by Kelpwatch^44^. Kelpwatch provides quarterly estimates of total emergent kelp canopy area (m^2^) within a selected geometry determined by the user. We downloaded total kelp area separately for our two study sites (TC and MR) from 2016 to 2023 for the third quarter (July–September). Since Kelpwatch only provides one kelp area value per quarter, in order to compare our kelp condition scores to the Kelpwatch canopy data, we took the mean kelp condition score for each site per year. We calculated the Spearman’s correlation coefficient to statistically compare the two methods.

### Species occurrence models

To investigate the temporal trends of each species over the eight years of the study, we modeled each species separately in relation to year and the day of year by site (MR and TC). We chose to fit generalized additive models (GAMs) as they allow for non-parametric fits to both continuous and categorical explanatory variables, while also accommodating complex distributions of response variables using a diversity of link functions. GAMs were fitted to the site values of species occurrence using the ‘mgcv’ R package (version 1.8-40;^45^), using a Restricted Maximum Likelihood approach (Table 1). The effect of year and day of year on species occurrence was modeled by site with penalized thin-plate regression splines with basis size limited to 3 to prevent overfitting ^46^. Variable selection was conducted with a shrinkage approach that penalizes non-significant variables to zero, hence selecting for more conservative models^47^.

**Table 1:** Summary of the temporal and trophic generalized additive models for each of the four species.

Model type	Model name	Model equation	Distribution	Link function
Temporal	Temporal urchin model	Urchin ~ year | site + DOY | site	Beta	Logit
	Temporal kelp model	Kelp ~ year | site + DOY | site	Tweedie	Log
	Temporal zooplankton model	Zooplankton ~ offset(log(n_stat)) + year | site + DOY | site	Tweedie	Log
	Temporal whale model	Gray whale ~ offset(log(effort)) + year | site + DOY | site	Tweedie	Log
Trophic path	Kelp path model	Kelp ~ urchin | site	Tweedie	Log
	Zooplankton path model	Zooplankton ~ offset(log(n_stat)) + kelp | site	Tweedie	Log
	Whale path model	Gray whale ~ offset(log(effort)) + kelp | site + log(zooplankton / n_stat) | site	Tweedie	Log

DOY = day of year; n_stat = number of stations sampled.

The response families and link functions were selected to accommodate the inherent differences in the statistical distributions of each species’ metric. The temporal urchin model was fit with a beta response distribution, epsilon parameter equal to 0.003 (half of the smallest positive urchin value recorded) and logit link function. The temporal kelp model was fit with a Tweedie distribution and log link function. The temporal zooplankton and whale models were fit with a Tweedie distribution, log link function, and an offset accounting for daily number of stations sampled within a site and time on survey effort, respectively (log-transformed).

To investigate the causal links between the four species in our study, we applied three separate GAMs representing our hypothesized trophic paths (Table 1) from (1) urchins to kelp (the kelp path model), (2) kelp as habitat to zooplankton (the zooplankton path model), and (3) kelp as an environmental cue and zooplankton as prey to whales (the whale path model). GAMs were run with the same methods and parameters as the temporal models, with the effect of each smooth term assessed separately by site and using a shrinkage approach implemented in the ‘mgcv’ R package to effectively perform variable selection. The shrinkage approach adds an extra penalty to each smoother and penalizes non-significant variables to zero^47^, alleviating the need to perform a stepwise regression to reveal significant species correlations. In the whale path model, we standardized the zooplankton site sum predictor metric by dividing it by the number of daily sampled stations, since the zooplankton abundance is directly affected by sampling effort. We log-transformed this value based on the knowledge that gray whales require a minimum threshold to initiate foraging behavior^30,48^, such that small variations at the lower end of the distribution may be more important to initiating gray whale foraging behavior in an area.

The descriptive performance of all models was assessed by examining the percent of deviance explained, which measures the discrepancy between the observations and the fitted values, using the likelihood of the model that varies depending on the distribution of the response and the link function (as computed by the ‘mgcv’ R package). The approximate significance of smooth terms is reported with χ^2^ values and associated *p*-values testing for a deviation of the smooth function from a flat or null function. Residuals were visually assessed to check for violations of model assumptions. Functional response plots were generated for each model to assess the effect of each smooth term upon species occurrence while all other variables are fixed to their mean. All analyses were performed using R (version 4.2.0; ^49^).

## Results

Across the eight-year study period (2016–2023), occurrence of urchins, kelp, zooplankton, and whales were assessed on 102 days in MR and 138 days in TC. The number of days sampled per year varied (2016 = 19, 2017 = 22, 2018 = 37, 2019 = 27, 2020 = 30, 2021 = 38, 2022 = 33, 2023 = 34). The species occurrence measurements conducted by the second analyst to test for robustness of methods showed a mean CV% of 3.44% (sd = 0.06, min = 0%, max = 26.6%) for urchin proportion, 4.61% (sd = 0.09, min = 0%, max = 28.3%) for kelp condition, and 0.06% (sd = 0.01, min = 0%, max = 20.2%) for zooplankton abundance, indicating that the second analyst produced comparable measurements.

**Figure 3:**
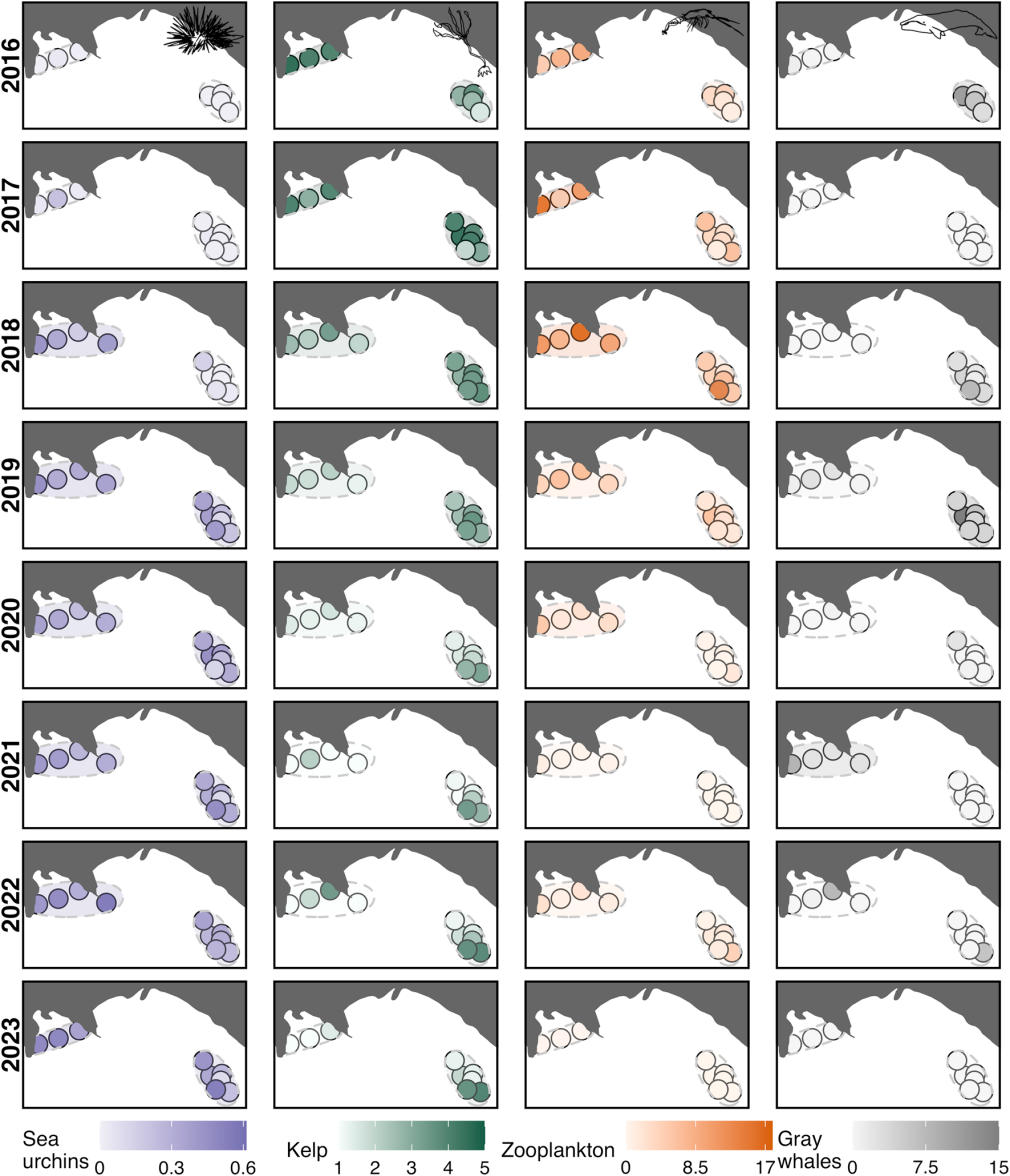
Mean station and site values per year (2016–2023) of relative purple sea urchin coverage, kelp condition, zooplankton abundance, and gray whale foraging time.

Despite some variability and inherent difference in scales across species, visual exploration of the data showed a spatial wave of species changes across the study area and temporal period (Fig. 3). Sampling stations in the western site of TC illustrated changes in the occurrence of urchins, kelp, zooplankton, and gray whales prior to stations in the eastern site of MR; increases in urchin percent cover and declines in kelp condition were detected in 2018 in TC, but not until 2019 in MR. Urchins were only present at two sampling stations at the start of the study period in 2016, but were seen at all stations by the final year.

**Figure 4:**
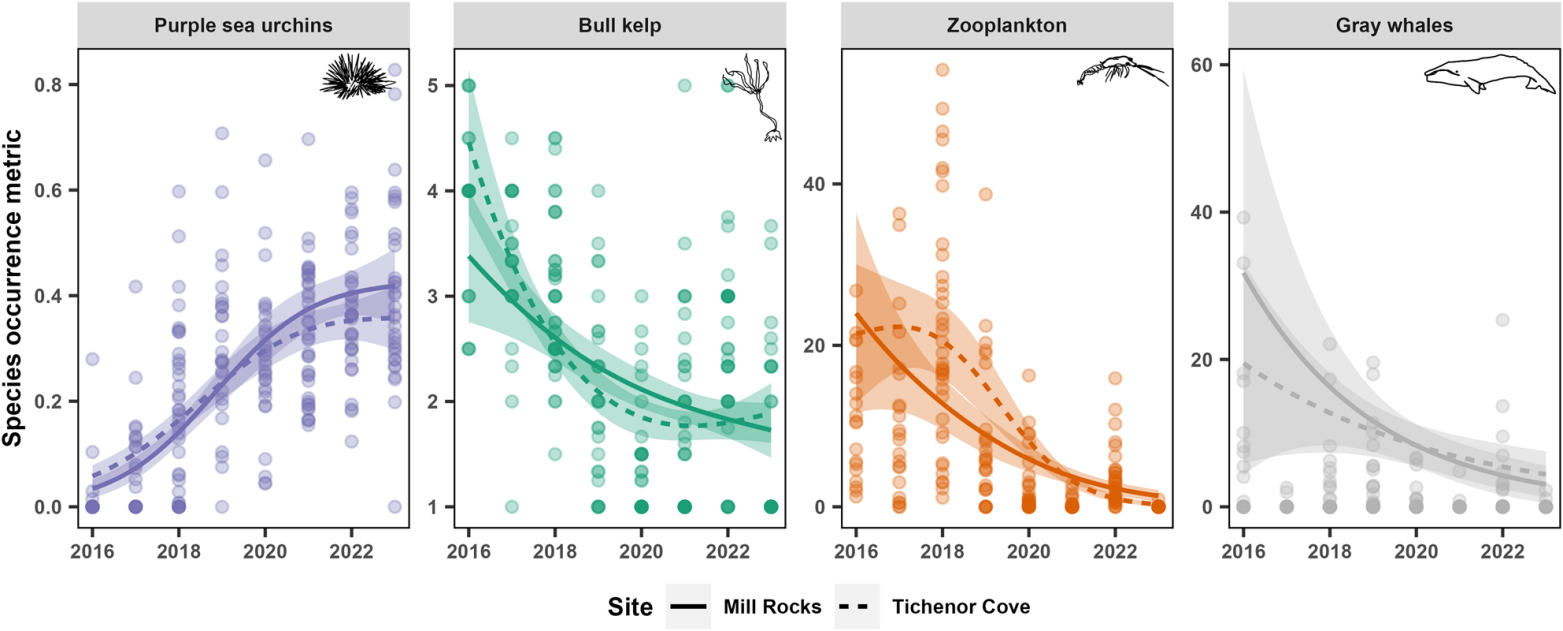
Temporal trends of purple sea urchin coverage, bull kelp condition, relative zooplankton abundance, and gray whale foraging time by year across the eight-year study period (2016–2023), from the generalized additive models. The colored ribbons represent approximate 95% confidence intervals. Line types represent the two study sites, Mill Rocks (MR; solid) and Tichenor Cove (TC; dashed). All curves are statistically significant (*P* < 0.05).

The temporal urchin model revealed that urchin coverage increased across the study period in both sites (Fig. 4), with a deviance explained of 60.3%. In contrast, kelp condition, zooplankton abundance, and gray whale foraging time all declined concurrently at both sites (Fig. 4), with deviance explained of 33.2%, 42.9% and 10.4%, respectively. Day of year did not have a significant effect in any of the temporal models (Table 2; see Fig. S5).

**Table 2:** Outputs of the temporal generalized additive models for each of the four species.

Model name	Response	Dev. exp. (%)	Smooth terms			
			Year		Day of year	
			MR	TC	MR	TC
Temporal urchin model	Urchin	60.3	χ^2^ = 106.1	χ^2^ = 96.6	χ^2^ = 0	χ^2^ = 0
			edf = 1.9	edf = 1.9	edf = 3.4e-5	edf = 1.8e-4
			***P* < 0.001**	***P* < 0.001**	*P* = 0.4	*P* = 0.9
Temporal kelp model	Kelp	33.2	χ^2^ = 13.6	χ^2^ = 43.4	χ^2^ = 0.7	χ^2^ = 1.0
			edf = 1.5	edf = 1.9	edf = 0.6	edf = 0.7
			***P* < 0.001**	***P* < 0.001**	*P* = 0.1	*P* = 0.09
Temporal zooplankton model	Zooplankton	42.9	χ^2^ = 26.2	χ^2^ = 68.3	χ^2^ = 0	χ^2^ = 0
			edf = 1.5	edf = 2.0	edf = 5.2e-5	edf = 4.8e-4
			***P* < 0.001**	***P* < 0.001**	*P* = 0.5	*P* = 0.7
Temporal whale model	Gray whale	10.4	χ^2^ = 4.4	χ^2^ = 2.8	χ^2^ = 0	χ^2^ = 0
			edf = 0.9	edf = 0.8	edf = 7.5e-5	edf = 1.0e-4
			***P* = 0.002**	***P* = 0.01**	*P* = 0.7	*P* = 0.8

Dev. exp. = deviance explained, χ^2^ = approximate significance of smooth term statistic, edf = estimated degrees of freedom, P = P-value.

Significant relationships with P-value < 0.05 are shown in bold.

**Figure 5:**
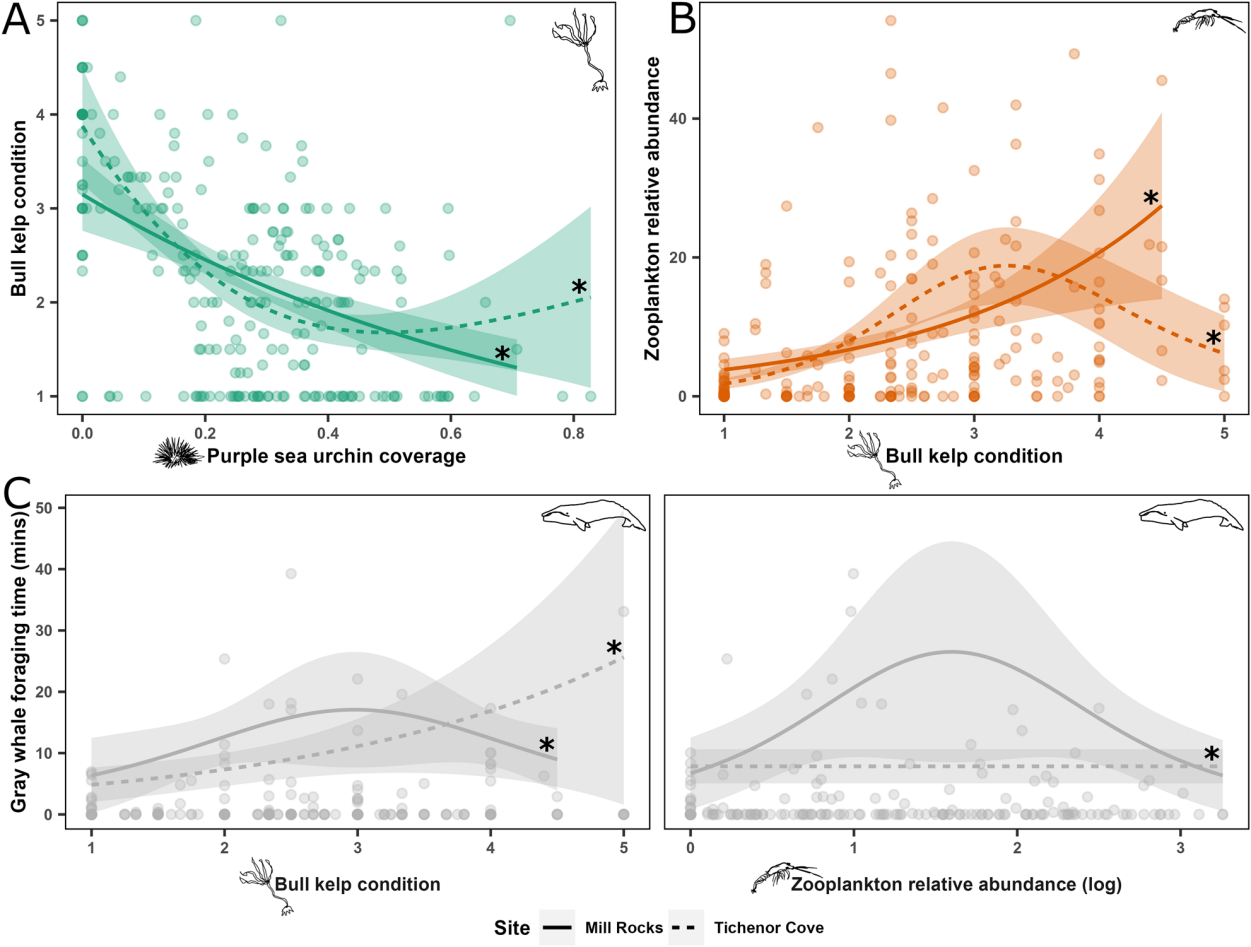
Effects derived from trophic path generalized additive models of purple sea urchin coverage on kelp condition (**A**), kelp condition on relative zooplankton abundance (**B**), and kelp condition and relative zooplankton abundance on gray whale foraging time (**C**). The colored ribbons represent approximate 95% confidence intervals. Line types represent the two study sites, Mill Rocks (MR; solid) and Tichenor Cove (TC; dashed). Curves with asterisks indicate statistically significant (*P* < 0.05) relationships.

Both the kelp path and the zooplankton path models aligned with hypothesized trophic relationships (Table 3). Urchin coverage had a significant negative correlation with kelp condition (30.4% deviance explained) in both sites (Fig. 5A). Kelp condition had a significant positive correlation with zooplankton abundance (21.8% deviance explained), with a strong positive trend in MR and a positive trend in TC until kelp condition score 3, after which the correlation leveled off (Fig. 5B). In the whale path model (11.6% deviance explained), zooplankton had a significant correlation with whales in MR whereby whale foraging time followed a bell-shaped trend as a function of zooplankton log-transformed abundance (Fig. 5C, right panel). Better kelp condition was also significantly correlated with greater whale foraging time in both sites (Fig. 5C, left panel; Table 3).

**Table 3:** Outputs of the trophic path generalized additive models for kelp, zooplankton, and gray whales.

Model name	Response	Dev. exp. (%)	Smooth terms					
			Urchin		Kelp		Zooplankton	
			MR	TC	MR	TC	MR	TC
Kelp path model	Kelp	30.4	χ^2^ = 13.8	χ^2^ = 25.8	–	–	–	–
			edf = 0.1	edf = 1.8				
			***P* < 0.001**	***P* < 0.001**				
Zooplankton path model	Zooplankton	21.8	–	–	χ^2^ = 11.1	χ^2^ = 27.5	–	–
					edf = 1.0	edf = 1.8		
					***P* < 0.001**	***P* < 0.001**		
Whale path model	Gray whale	11.6	–	–	χ^2^ = 1.1	χ^2^ = 3.0	χ^2^ = 2.5	χ^2^ = 0
					edf = 0.7	edf = 0.9	edf = 0.8	edf = 1.8e-4
					***P* = 0.01**	***P* = 0.009**	***P* = 0.01**	P = 0.7

Dev. exp. = deviance explained, χ^2^ = approximate significance of smooth term statistic, edf = estimated degrees of freedom, *P* = *P*-value. Significant relationships with *P*-value < 0.05 are shown in bold.

Relationships not included in the models are represented by (–).

## Discussion

In this study, we observed concurrent spatio-temporal patterns of zooplankton decline with an urchin-kelp trophic cascade, as well as a decline in gray whale foraging time in the study area over eight years. While we did not document sunflower sea star decline in our study, we posit that the total loss of this sea urchin predator (^22^ documented 100% decline in this same area in 2015, one year prior to our study) instigated the rise in purple sea urchin populations in our study sites as urchins were released from predation pressure. Concurrently, warm water and low nutrient conditions brought about by the MHW from 2014 to 2016 depressed kelp growth and recruitment, resulting in less detrital drift kelp for urchins to passively feed on. Thus, increasing urchin populations that switched from passive to active herbivory to satiate their hunger, caused declines in kelp condition and, ultimately, kelp density. Our temporal and trophic path models indicate that as urchin coverage increased, kelp condition decreased, thus likely reducing available good quality habitat for zooplankton that also declined in abundance (Fig. 4). Concurrently, gray whale foraging time in Port Orford decreased together with a statistical correlation with zooplankton abundance though only at one site (MR) and following a non-linear trend, which may be due to fine-scale mismatches between patchy prey and mobile predators^50–52^. The whale path model also revealed statistically significant correlations between increased whale foraging time and improved kelp condition, suggesting that multi-species connections across this trophic cascade exist under certain conditions.

As hypothesized, urchins had a significant negative correlation with kelp condition in both sites (Fig. 5A) and these species showed opposite temporal trends across the study period (Fig. 4). These findings provide evidence that a phase shift from a kelp forest to an urchin barren may have occurred in our study region during our study period, similar to events documented in geographically-adjacent northern California^17,23^. Sunflower sea star populations have not recovered from SSWD between Baja California, Mexico to Cape Flattery, Washington, U.S.A. and have been deemed functionally extinct in this region^53^, which raises concerns for the health of the reef ecosystem in our study area given that sunflower sea stars were the only predator of urchins. The simultaneous decline of zooplankton abundance across the 8-year study period (Fig. 4) suggests that the documented urchin-kelp dynamics may have cascading indirect effects to zooplankton. Furthermore, kelp condition had a significant positive correlation with zooplankton in both sites in the zooplankton path model (Fig. 5B), which supports our hypothesis that kelp forests are an important habitat for zooplankton in this nearshore environment as they provide shelter^32–34^ and food resources^31^. Interestingly, while this relationship was linear in TC, it was bell-shaped in MR, suggesting that other factors besides healthy bull kelp contribute to high zooplankton abundances. Mysids, which composed ~85% of the zooplankton community in our study area across the study period (*Torres, unpublished data*) and are the predominant zooplankton consumed by PCFG gray whales in their feeding range, are spatially dynamic and inherently patchy^54^, and their abundance and retention are also a function of upwelling^55^, tides, currents^33,34,56^, habitat structure, and presence of perennial understory kelps, which we were unable to account for in this study.

While we did detect a significant correlation between zooplankton and whale foraging time in MR, the trend is bell-shaped with wide confidence intervals, making it difficult to interpret. Furthermore, we found no correlation between zooplankton and whale foraging time in TC, indicating that there are clearly other confounding factors at play in this system. These results are surprising, considering that zooplankton is the primary prey for gray whales foraging in this region and could be caused by a mismatch between whale behavior and zooplankton at the fine spatial scale of this study, as often observed when trying to spatially link predators and their prey (e.g.^50–52^). Differences in sampling effort (prey kayak sampling only occurred once a day whereas gray whale tracking was continuous throughout the day) could also cause a temporal mismatch and did not allow us to account for gray whale consumption of prey. Additionally, PCFG gray whales make trade-offs between prey quantity and quality while foraging^30^, which we were unable to account for in this study as prey quality data (i.e., caloric content) was not collected in all sampling years. Despite the unclear effect of zooplankton on whales, there was a significant correlation between kelp and whales in both sites, whereby in MR whale foraging time was lower when kelp was totally absent compared to when it was present but partially damaged, and in TC whale foraging time increased linearly with improved kelp condition (Table 3). This finding corroborates prior correlative findings between gray whale foraging behavior and rocky reefs with kelp across the PCFG range^26,29,30,36^. We postulate that since kelp habitat may aggregate or retain zooplankton prey^32^, gray whales may use kelp as an environmental cue to locate prey patches. Thus, even when some very small abundances of zooplankton were present, whales may not have visited our study sites due to the lack of kelp. While the mechanism remains unknown, our findings emphasize the importance of healthy reef and kelp forests to PCFG gray whales. Across broader spatiotemporal scales, gray whale population size and body condition are responsive to variation in ocean conditions and resource availability^57,58^. Thus, we propose a mechanistic driver for the observed reduced body condition in PCFG gray whales^37,38^ that could be investigated in future research: decreased zooplankton prey availability due to kelp habitat degradation as a result of increased urchin density and other environmental factors.

We acknowledge that our data processing methods for assessing kelp and urchin occurrence were non-traditional, yet we believe that our methods effectively document real trends that describe the dynamics between urchins, kelp, zooplankton, and gray whales. To build upon our findings and test our hypothesized mechanistic links between the four species, all components of this complex trophic system, including gray whales and their body condition, should be monitored over a longer time period to capture multiple phase shifts and use more traditional field methods to assess absolute urchin and kelp density, such as scuba surveys, if possible. Furthermore, monitoring a control area where urchin predators exist alongside the other trophic levels documented in our study would further help to test our hypothesized mechanistic links. Despite being non-traditional, our method of qualitatively assessing kelp condition is valuable as changes in health can be a precursor to changes in density or abundance in ecological systems^57,59^. By monitoring kelp health, negative effects on kelp ecosystems may be detected before changes in species abundance or density occur. Future research should explicitly investigate the links between kelp health and density as measured *in situ* and through satellite remote sensing (e.g.^60,61^). Given expected increased frequency and intensity of MHWs^62^, potential sea otter reintroduction to Oregon waters^39^ and the continued lack of sunflower sea star recovery^53^, such monitoring effort is particularly important as these overarching factors will impact the dynamics of this complex trophic system. It is possible that the temporal trends and correlations we observed in our study, particularly of zooplankton and kelp, may be in response to environmental changes, such as the MHW, water temperature or nutrient circulation. Yet, despite temperature anomalies returning to near normal distributions in 2016^63^, we documented a continued progression in kelp condition and zooplankton abundance decline, concurrently with a rise in urchin coverage across the rest of the study period, suggesting that more than environmental conditions were at play^23^. Nevertheless, the impact and interplay of environmental changes on these trophic dynamics warrants further investigation.

Although we tested our hypothesis in a small area (~ 12 km^2^) relative to the full extent of the PCFG range, trophic dynamics documented in our study system may be representative of patterns across their range given ecological and topographic similarities in habitat use patterns^30,36,64,65^. If these dynamics occurred in other locations along the northeast coast of the Pacific, the decline of zooplankton abundance and gray whale foraging time could raise concerns for the health of the reef ecosystem more broadly. Zooplankton, particularly mysids, are the primary prey for a number of fish species of recreational and commercial importance, such as groundfish and salmon^66^, which may suffer nutritional or abundance declines with the loss of kelp and zooplankton. Additionally, baleen whales play a critical role in nutrient cycling^67,68^ as they increase primary productivity through defecations that transport nutrients both vertically and horizontally through the water column^69^. After whaling reduced populations in the Southern Ocean, baleen whales recycled one order of magnitude less iron than pre-whaling populations^70^. A localized decline of gray whale foraging time in nearshore, reef habitats, such as in our study system, may reduce their nutrient contribution to the ecosystem, thus intensifying already low nutrient conditions caused by the MHW, and further hinder kelp development and growth. These cumulative impacts on the ecosystem may promote an alternative stable sea urchin state rather than a reversal back to a healthy kelp forest phase^24^, especially given the lack of recovery by sunflower sea stars^53^. While the mechanisms are still unclear, our study suggests that a consumer-mediated trophic cascade may indirectly affect predators via impacts on their prey. Spatio-temporal correlations across eight years point to undocumented indirect effects caused by an urchin-kelp forest trophic cascade on two species (zooplankton and gray whales). We propose that further research is conducted in this complex ecosystem under the postulation that environmental change may exacerbate and expand impacts of trophic cascades across more species and interspecies linkages than previously thought.

### Supplementary Information

Supplementary Information.

## Data Availability

Data, code (10.6084/m9.figshare.20419398) and supplemental videos (10.6084/m9.figshare.20419824) are provided via the Figshare data repository.
