# Supplementary material for: Exploring indirect effects of a classic trophic cascade between urchins and kelp on zooplankton and whales
Source: Sci Rep. 2024 Apr 29;14:9815. doi: 10.1038/s41598-024-59964-x (PMC11059377; doi:10.1038/s41598-024-59964-x)
Supplement: Supplementary file 1 — Supplementary Information. [file 41598_2024_59964_MOESM1_ESM.docx]

**Supplementary information**

**Authors:** Lisa Hildebrand, Solène Derville, Ines Hildebrand, Leigh G. Torres

**Manuscript title:** Exploring indirect effects of a classic trophic cascade between urchins and kelp on zooplankton and whales

**Journal Name:** Scientific Reports


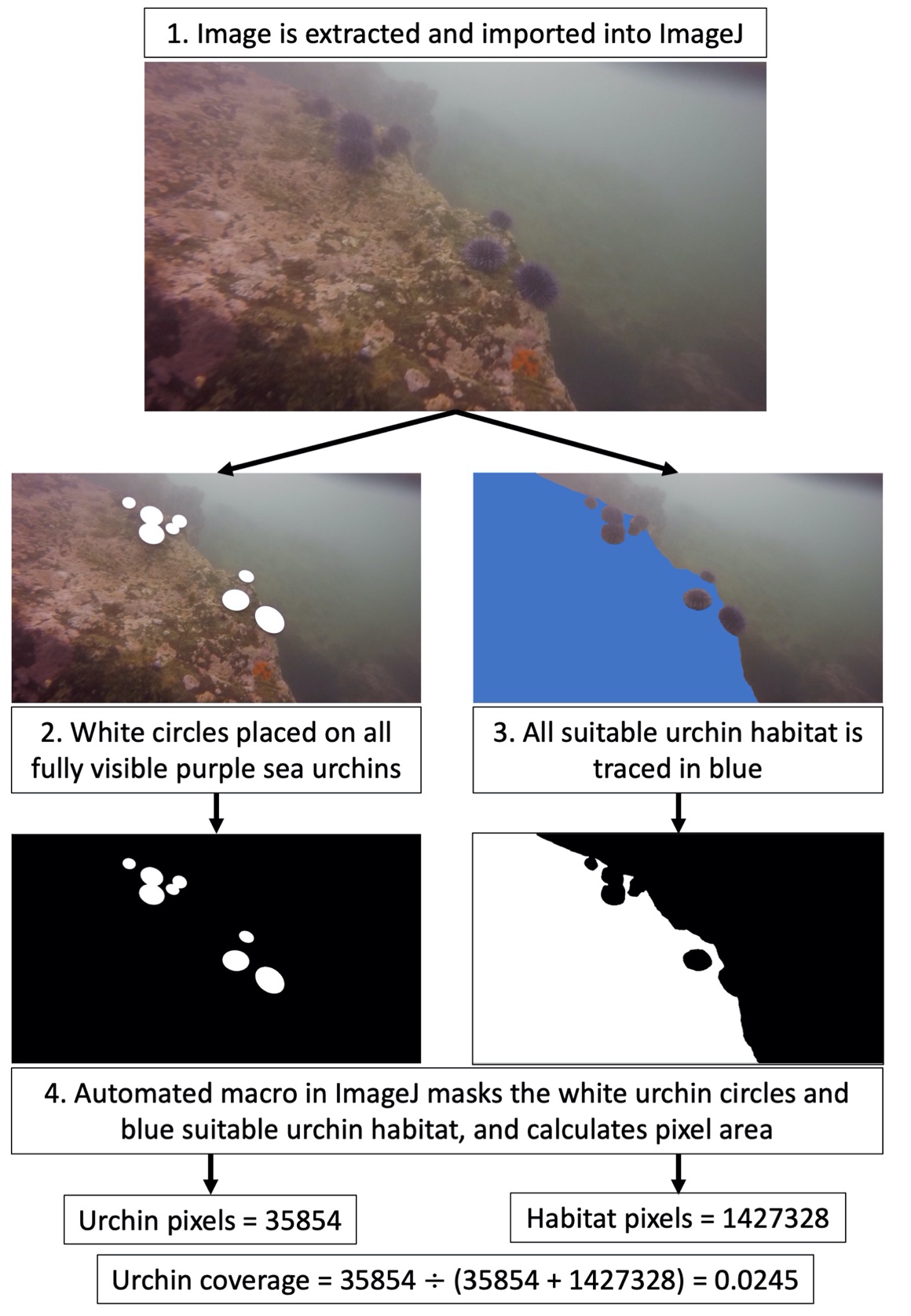


**Figure S1.** Workflow of urchin coverage method from image extraction to processing in ImageJ to quantify urchin coverage in pixel area.


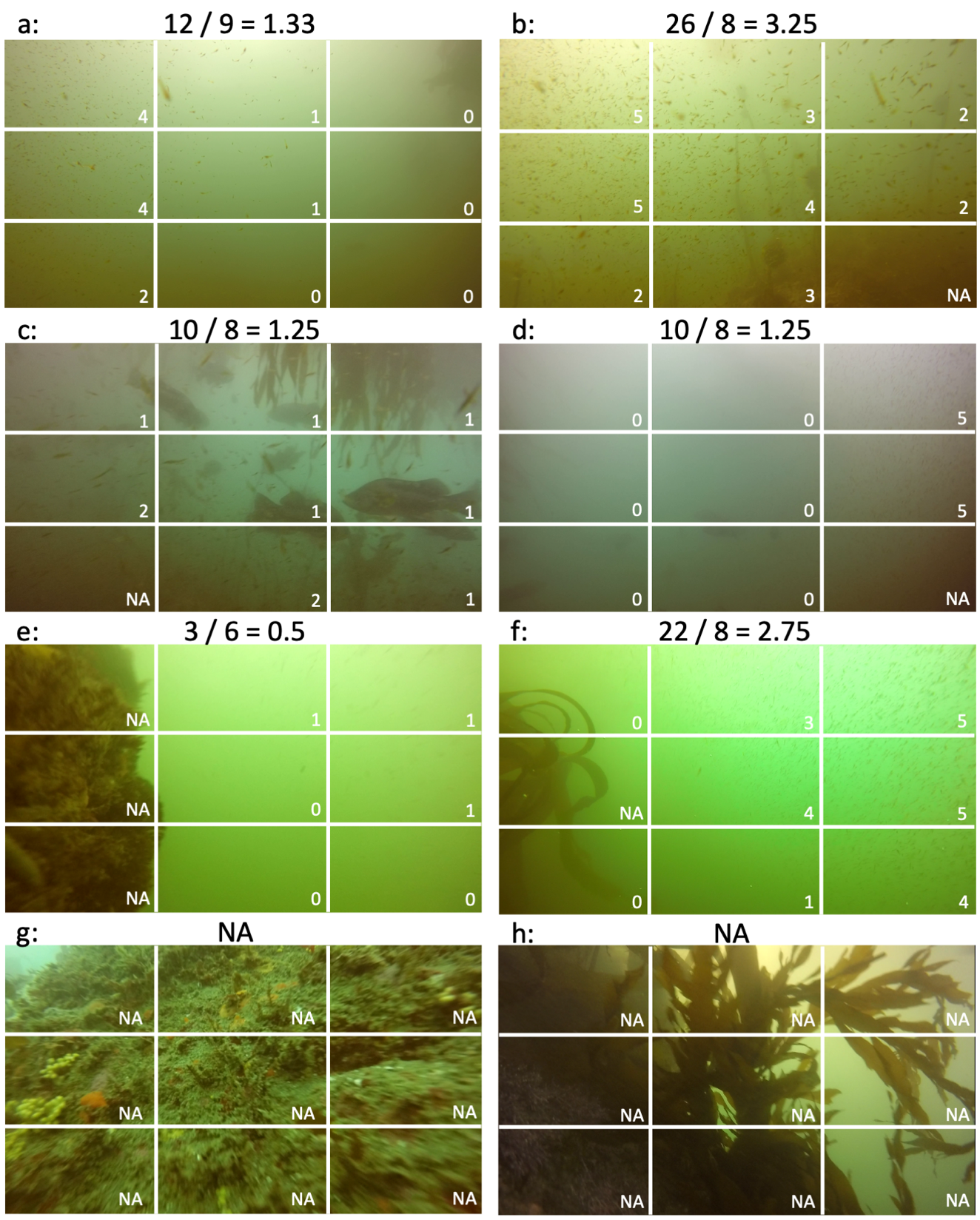


**Figure S2. (a-h)** Examples of GoPro video still images with overlaid 3x3 grid used to score images for relative zooplankton abundance estimation. Grid cells have been assigned classification scores of relative zooplankton abundance (0-5 and NA). The different images represent different examples of clarity and obstruction that were observed from GoPro videos. Above each image is the mean relative zooplankton abundance calculated for each still image as it is the sum of all numeric scores divided by the total number of grid cells (excluding NAs). Taken from [1].


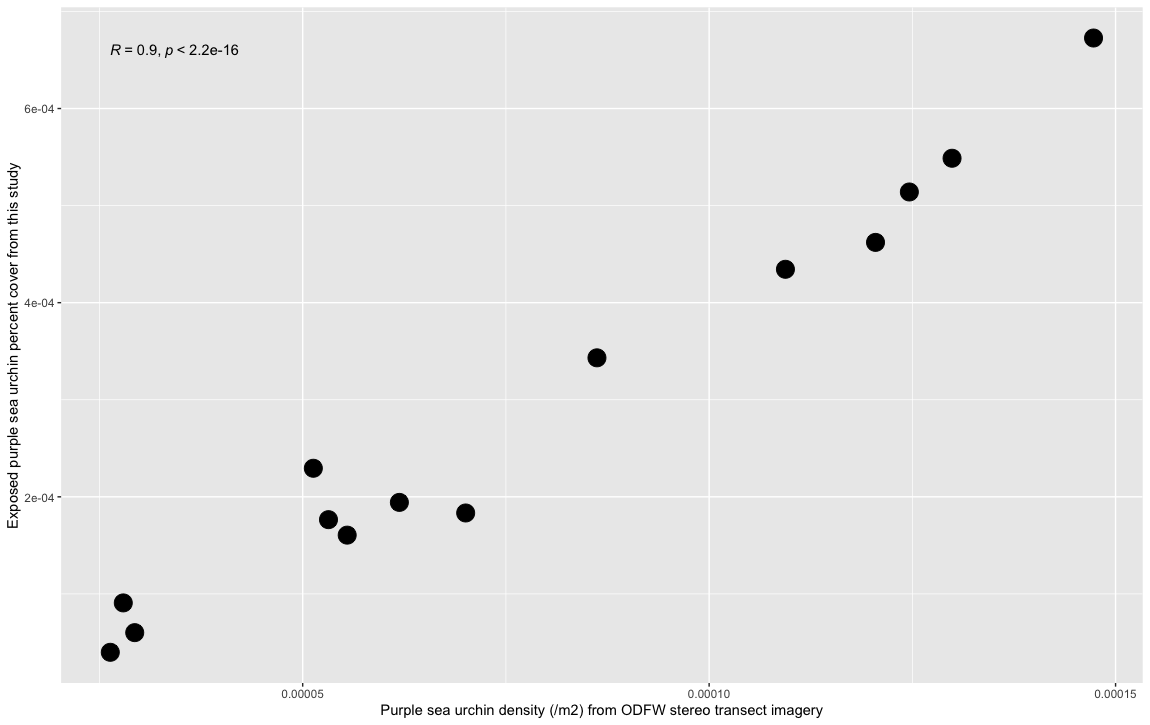


**Figure S3.** Comparison of the *in-situ* exposed purple sea urchin percent cover method from this study to purple sea urchin density (/m^2^) from the Oregon Department of Fish and Wildlife (ODFW) stereo video transect imagery.


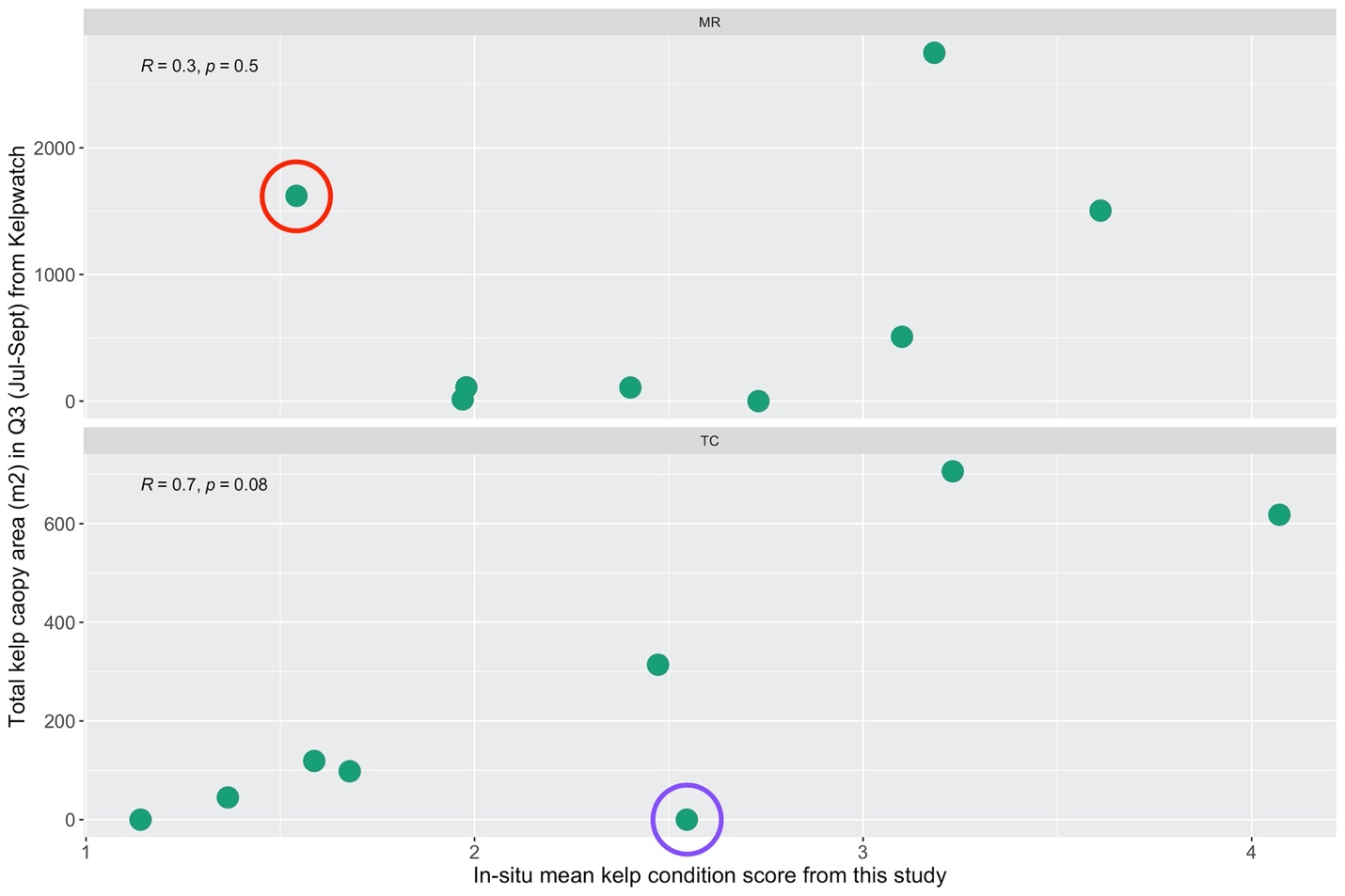


**Figure S4.** Comparison of total emergent kelp canopy area (m^2^) derived from Landsat imagery from Kelpwatch.org [2] to the *in-situ* mean kelp condition score derived from this study. Points circled in red and purple are considered outliers due to Kelpwatch data discrepancies and are further discussed in Text S2.

**
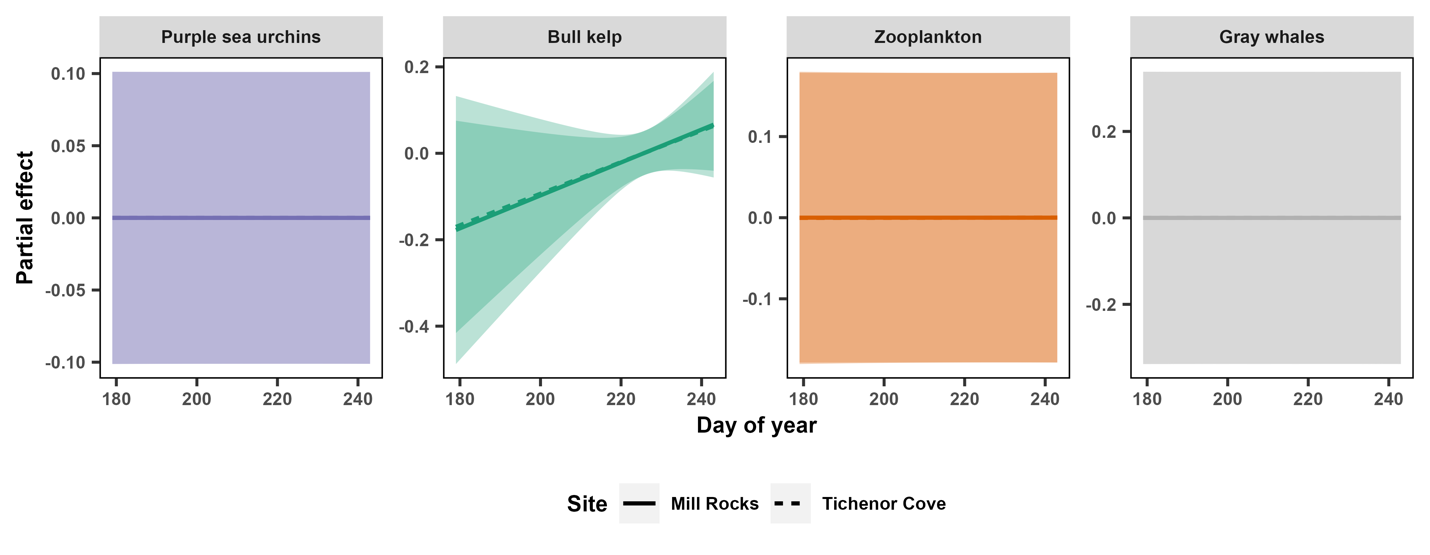
**

**Figure S5.** Temporal trends of purple sea urchin proportion, bull kelp condition, relative zooplankton abundance, and gray whale foraging time by day of year across the eight-year study period (2016-2023), from the generalized additive models. The colored ribbons represent approximate 95% confidence intervals. Line types represent the two study sites, Mill Rocks (MR; solid) and Tichenor Cove (TC; dashed).


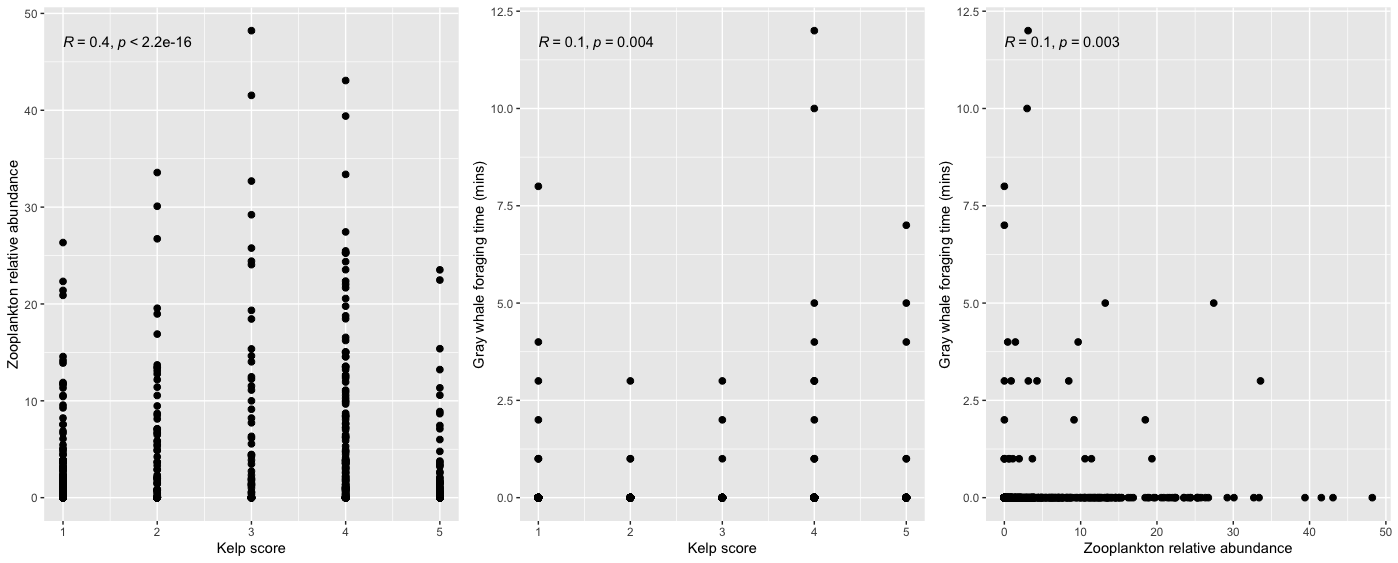


**Figure S6.** Correlations between raw occurrence data for the variables included in the full whale trophic path model.

**Text S1. Explanation of stereo imagery collection by the Oregon Department of Fish and Wildlife**

The Oregon Department of Fish and Wildlife (ODFW) collected stereo video imagery along a 30-m transect at Orford Reef, offshore of our study site, using a remotely operated vehicle. The stereo video system consisted of two GoPro Hero4 cameras in custom Sexton housings mounted on a fixed plate with 18 cm baseline separation, both pointing forward with 20° downward tilt below horizontal. The system was pre-calibrated and the video was processed using SeaGIS software, which provides highly accurate measurements of the position of objects within the field of view of both cameras. The video transect was segmented into “virtual quadrats”, which were established and measured by defining four 3D points (the four corners of the quadrat) using visually distinct points on the seafloor in the image. Within each virtual quadrat, the manual count of purple sea urchins was divided by the quadrat area to derive urchin density (m^-2^).

**Text S2. Validation results of non-traditional methods**

Exposed purple sea urchin percent cover

The comparison of our exposed purple sea urchin percent cover method to ODFW’s purple sea urchin densities revealed a strong positive correlation (Fig. S3; Spearman’s, *r*=0.9, *p*<0.001). Therefore, we believe our urchin pixel density method to derive purple sea urchin percent cover is able to accurately capture changes in urchin density in our study area.

Kelp condition

The comparison of our mean kelp condition scores to the quarterly total kelp canopy area (m2) from Kelpwatch showed a strong positive correlation in TC (Fig. S4, Spearman’s, r=0.7, p=0.08) and a weak positive correlation in MR (Fig. S4, Spearman’s, r=0.3, p=0.5), though both were non-significant. Non-significance appears to occur due to a couple large outliers (Fig. S4), which we believe is due to discrepancies in scale between the Kelpwatch data and our kelp metrics. First, in order to average out the effects of currents and tides, Kelpwatch creates seasonal/quarterly averages, which means that the quarter in which our data collection occurs (July-August) also encompasses data from September (often the month with peak kelp canopy area of the year). This would explain why when our in-situ kelp condition value is low, the corresponding Kelpwatch value is much higher (see point circled in red in Fig. S4). Secondly, Kelpwatch processes Landsat satellite imagery for its kelp canopy area which has a resolution of 30 m per grid cell. This resolution is quite coarse relative to the scale we are investigating in Port Orford. Furthermore, the Kelpwatch team acknowledges the following on their Methodology page: “*these data are well-suited to investigate kelp dynamics at a larger spatial scale, but that local-scale application might require higher-resolution data in order to detect sparse and/or fringing kelp [3, 4]. For example, areas very close to shore or offshore rocks (typically within one Landsat pixel; ~30m) or areas that are exposed during low tide will be removed by the land masking procedure and thus the kelp canopy area within these pixels will not be measured. Additionally, areas with sparse kelp canopy may not be detected by the classifier.*”. This would explain why when our in-situ kelp condition value is high, the corresponding Kelpwatch value is much lower (see point circled in purple in Fig. S4). If these two major outliers were removed from the dataset, then the correlation in MR would become significant (Spearman’s, r=0.9, p=0.007) and the correlation in TC would become stronger (Spearman’s, r=0.7, p=0.09). Given that the Kelpwatch data is an average across three months (July-September), we do not expect that our in-situ kelp metric averaged across 4-6 weeks (e.g., 1/3-1/2 the length of the Kelpwatch period) will perfectly align. Furthermore, the coarser scale of the Kelpwatch data compared to our very fine-scale, nearshore measurements, also contribute to imperfect comparison. Nevertheless, despite these differences, we believe that the data match well enough to validate our kelp condition metric and also allows us to make the argument that declining kelp condition in terms of frond and stalk health, is a relatively good proxy for declining kelp canopy.

**References**

1. Hildebrand, L., Sullivan, F.A., Orben, R.A., Derville, S., and Torres, L.G. (2022). Trade-offs in prey quantity and quality in gray whale foraging. *Mar. Ecol. Prog. Ser.* 695, 189-201.
2. Bell, T., Cavanaugh, K. & Siegel, D. (2023). SBC LTER: Time series of quarterly NetCDF files of kelp biomass in the canopy from Landsat 5, 7 and 8, since 1984 (ongoing) ver 22. Environmental Data Initiative [https://doi.org/10.6073/pasta/da60200f3f71a5bde3f469cc72d47b4d. Accessed 2023-12-01](https://doi.org/10.6073/pasta/da60200f3f71a5bde3f469cc72d47b4d.%20Accessed%202023-12-01).
3. Hamilton, S. L., Bell, T. W., Watson, J. R., Grorud-Colvert, K. A. & B. A., Menge. (2020). Remote sensing: generation of long-term kelp bed data sets for evaluation of impacts of climatic variation. *Ecology*. 101(7); 10.1002/ecy.3031.
4. Saccomanno, V. R., *et al.* (2022). Using unoccupied aerial vehicles to map and monitor changes in emergent kelp canopy after an ecological regime shift. *Remote Sensing in Ecology and Conservation*. 9(1), 62-75.
